# Proximal row carpectomy with interposition arthroplasty using both capsular flap and acellular human dermal matrix

**DOI:** 10.1186/s12891-024-07305-4

**Published:** 2024-03-05

**Authors:** Dae-Hee Lee, Joong-Bae Seo, Jae-Uk Jung, Jae-Sung Yoo

**Affiliations:** Department of Orthopaedic Surgery, Dankook University College of Medicine, Manghyangro 201, Dongnam-gu, Cheonan, 330-715 Chungnam Korea

**Keywords:** Wrist arthritis, Proximal row carpectomy, Interposition arthroplasty, Acellular dermal matrix

## Abstract

**Background:**

In cases of wrist arthritis, proximal row carpectomy (PRC) has been widely utilized and shown favorable long-term outcomes. However, its applicability is limited in cases where arthritis extends to the lunate fossa or capitate. Recently, surgical approaches combining various methods of interposition arthroplasty have been introduced to overcome these drawbacks. The purpose of this study was to perform PRC and interposition arthroplasty with dorsal capsule and acellular dermal matrix(ADM),and analyze the clinical outcomes of these procedures.

**Methods:**

Fourteen cases who underwent PRC and interposition arthroplasty using both dorsal capsular flap and ADM were retrospectively recruited. The researchers assessed the patients’ Visual Analog Scale (VAS) pain score, Disabilities of the Arm, Shoulder and Hand (DASH) scores, range of motion (ROM), retear, and radiocarpal distance (RCD).

**Results:**

One year post-surgery, both the VAS pain scores, DASH scores, and ROM showed statistically significant improvement compared to before the surgery. Upon reviewing the radiological results, the postoperative mean RCD was 4.8 ± 0.8 mm and one year follow up mean RCD was 3.6 ± 0.5 mm at one year post-surgery. Moreover, in the one year follow-up, there was no observed failure of the allodermis graft in any of the cases.

**Conclusion:**

The PRC and interposition arthroplasty with ADM demonstrated significantly improved clinical outcomes after surgery, showing a maintain of RCD without graft failure effectively.

## Introduction

Proximal row carpectomy (PRC) provides a beneficial surgical alternative for addressing wrist arthritis. PRC has demonstrated efficacy in treating arthropathy resulting from scapholunate advanced collapse, scaphoid nonunion advanced collapse, Keinböck’s disease, and the aftermath of perilunate dislocations [1–3]. 

PRC is frequently juxtaposed with partial wrist arthrodesis involving scaphoid excision and four-corner fusion, given their analogous indications and favorable long-term outcomes [1, 4]. 

Factors related to the patient, such as a younger age and occupation involving manual labor, tend to favor partial wrist arthrodesis over PRC. Studies have indicated that partial wrist arthrodesis is more effective in preserving wrist strength than PRC in such cases. In situations where motion-preserving alternatives and other treatments prove ineffective, total wrist fusion serves as a final option in salvage surgery [2, 5]. 

Degenerative changes at the lunate fossa or capitate represent a significant limitation for PRC. A PRC with a capsular interposition flap between the radiocapitate articulation has been recently described with satisfactory results among 8 wrists with a mean follow up of 41 months [5]. However, the use of a capsular interpositioning flap is limited in cases where there is insufficient tissue due to advanced progression of the radiocarpal joint. Recently, to overcome these limitations, satisfactory results have been reported with the use of acellular human dermal matrix (ADM) for interposition arthroplasty in cases where the constraints were significant due to the advanced state of the radiocarpal joint [6, 7]. 

Therefore, the authors conducted PRC with interposition arthroplasty using both dorsal capsular flap and ADM, aiming to report on the clinical outcomes of this approach.

## Method

After obtaining Institutional Review Board of our institution approval, the study focused on a total of 14 patients among 17 patients who underwent PRC (Proximal Row Carpectomy) with interposition arthroplasty, involving the use of both dorsal capsular flap and ADM (Acellular Dermal Matrix). Two patients were excluded from the analysis due to loss of follow-up, and one patient who underwent simultaneous Sauve-Kapanji procedure for concomitant distal radioulnar joint arthritis was also excluded. (July 2020 - December 2022).

The study included patients with stage 3 or 4 scapholunate advanced collapse (SLAC), stage 2 or 3 scaphoid nonunion advanced collapse (SNAC), and those with arthritis progressing in the capitate head or lunate facet.

### Surgical technique

**Fig. 1:**
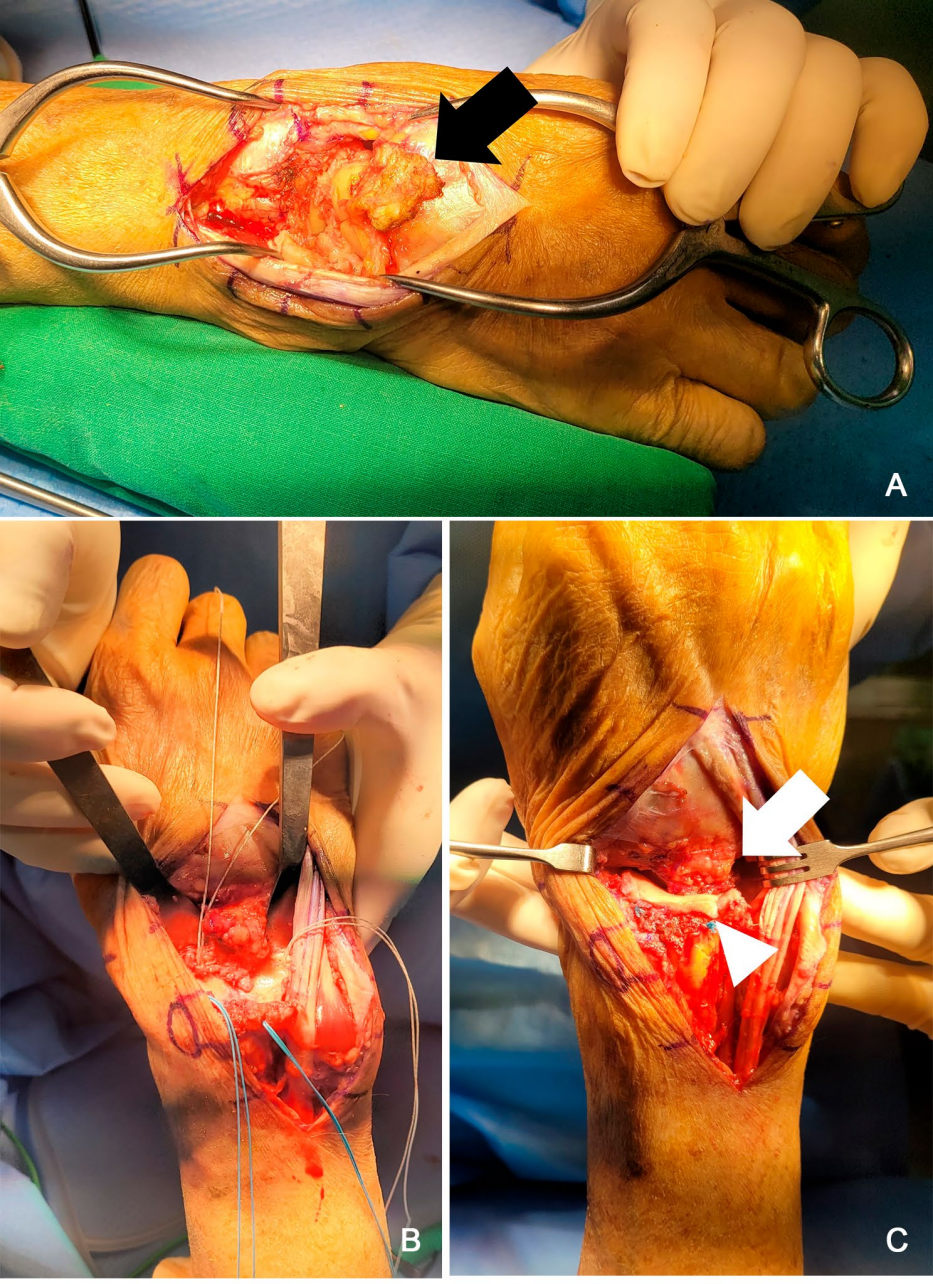
Images displaying the procedural method of proximal row carpectomy with interposition arthroplasty using capsular and dermal allografts. (**A**) The dorsal capsule (black arrow), which is based proximally, is lifted from the carpometacarpal joints to the distal articular boundary of the radius. (**B**) Four all-suture anchors were inserted into the articular margin of the radius. (**C**) Dermal allograft interposition (arrowheads) was carried out at each corner using four suture anchors. Subsequently, dorsal capsular interposition arthroplasty (arrow) was performed

Under regional anesthesia and via a dorsal midline cut, the extensor retinaculum was recognized and pivoted open from the second to the fifth extensor compartments. Following the withdrawal of the extensors, a capsular flap with a proximal origin was lifted from just above the second and fourth carpometacarpal joints to the distal articular edge of the radius (Fig. 1A). The lunate, triquetrum, and scaphoid were then gradually extracted using a ronguer, guided by a 1.8 mm Kirschner wire functioning as a joystick. After inserting 4 to 6 Q fix all-suture anchors (Smith & Nephew, London, United Kingdom) into the distal radius (Fig. 1B), each corner was secured by attaching acellular human dermal matrix (BellaCell; Hans Biomed Corporation, Daejeon, Korea) and sutures. Finally, capsular interposition was implemented by suturing the radial end of the capsule to the radioscaphocapitate ligament and the ulnar end to the ulnocarpal ligament (Fig. 1C).

### Postoperative rehabilitation

After surgery, a short arm splint was applied for four weeks. Finger movement was allowed immediately after the surgery. Wrist passive and active exercises, as well as daily activities, were permitted starting four weeks post-surgery. Weight-bearing tasks were allowed after 12 weeks post-surgery.

### Clinical and radiological evaluation

Before the surgery and at one year post-surgery, the researchers assessed the patients’ Visual Analog Scale (VAS) pain score, Disabilities of the Arm, Shoulder and Hand (DASH) scores, and range of motion (ROM).

Postoperative surgery and at one year post-surgery, plain radiography was used to measure the radiocarpal distance (RCD). The outcomes were measured through assessments performed by an orthopedic specialist (J.W.J.), and Radiological evaluations were conducted with measurements taken twice by two observers, and the mean values were used for analysis (D.H.L. and J.W.J.).

## Results

Fourteen cases were recruited and observed for a minimum of 1 year. The average age was 65.5 ± 7.5 years, with a gender distribution of 8 males and 6 females. The dominant arm occurred 5 cases, and the non-dominant arm occurred 9 cases (Table 1).

**Table 1:** Demographic data

Variable	Combination PRC and interposition arthroplasty (*n* = 14)
Mean age	65.5 ± 7.5
Gender (Male: Female)	8 : 6
Dominant arm: Non-dominant arm	6 : 8
Height (cm)	167.2 ± 12.3
Weight (kg)	66.3 ± 10.3
Body mass index	23.7 ± 3.6
Smoking: Non-smoking	4 : 10
ASA class (1:2:3)	11 : 3 : 0
Mean follow-up (month)	15.6 ± 4.8

ASA; American Society of Anesthesiologists

**Fig. 2:**
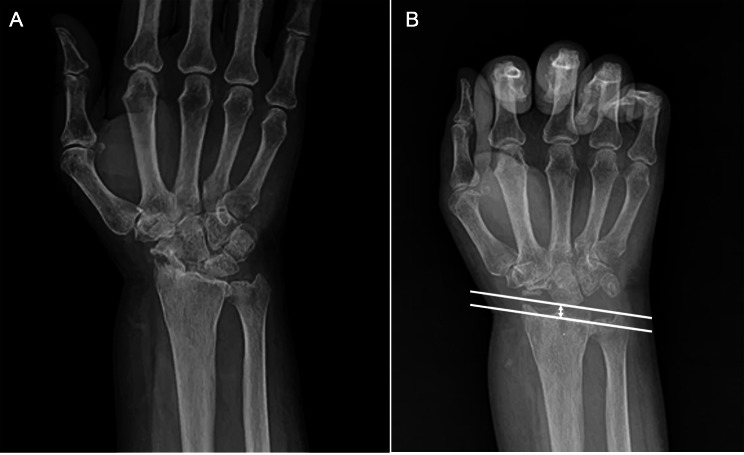
Simple radiography of 67-year-old female who underwent proximal row carpectomy with interposition arthroplasty utilizing both a capsular flap and acellular human dermal matrix (**A**) Radiographic images taken before the surgery on the patient’s wrist revealed significant arthritic alterations in the lunocapitate and radiolunate joints, along with a collapsed scaphoid. (**B**) On radiographic examination one year post-surgery, it was observed that the radiocarpal distance was well maintained

The VAS pain score significantly improved from 7.7 ± 2.8 preoperatively to 1.7 ± 0.7 postoperatively. The DSAH score showed a notable improvement, decreasing from 66.1 ± 17.4 to 16.8 ± 10.9 after surgery. Flexion-Extension ROM increased from 64.2 ± 16.1 preoperatively to 72.7 ± 14.8 postoperatively, while Pronation-Supination ROM improved from 123.8 ± 15.7 preoperatively to 137.7 ± 18.5 postoperatively. These changes were statistically significant, indicating overall improvement (Table 2). After surgery, the RCD was 4.8 mm with a standard deviation of ± 0.8 mm. One year after surgery, the RCD was 3.6 mm with a standard deviation of ± 0.5 mm (Fig. 2).

**Table 2:** Clinical outcomes

Variable	Preoperative	Final follow-up	*p*-value
VAS pain score	7.7 ± 2.8	1.7 ± 0.7	< 0.001
DASH score	66.1 ± 17.4	16.8 ± 10.9	< 0.001
Flexion Extension ROM	64.2 ± 16.1	72.7 ± 14.8	0.02
Pronation Supination ROM	123.8 ± 15.7	137.7 ± 18.5	0.003

VAS: Visual Analog Scales, DASH: Disabilities of the Arm, Shoulder and Hand, ROM: Range of Motion

## Discussion

While positive outcomes have been documented following proximal row carpectomy, additional research suggests that extensive cartilage deterioration on both sides of the radiocapitate joint is prone to result in an unfavorable prognosis [1, 8–10]. Salomon and Eaton addressed these limitations by performing a combination of PRC and dorsal capsular interposition in a study involving 12 patients, reporting satisfactory outcomes with a flexion-extension range of motion of 94 degrees and pain relief [11]. In 2009, Kwon et al. also reported satisfactory results with a significant pain relief and an average flexion-extension ROM of 71.9 degrees after performing a combination of PRC and dorsal capsular interposition surgery [5]. 

However, using only dorsal capsule interposition may not be sufficient to prevent adequate bone-to-bone contact in the radiocarpal joint, as there are cases where the amount or thickness of tissue is not enough. Therefore, recently, interposition arthroplasty using Dermal Allograft has been proposed as an alternative. In 2011, Carneiro et al. [12], and in 2018, Rabinovich and Lee [7] introduced the surgical technique of interposition arthroplasty using PRC and dermal allograft. In 2020, Lee et al. reported improved outcomes, including a flexion-extension ROM of 113 degrees, pronation supination ROM of 170.5 degrees, and a DASH score improving from 63.5 before surgery to 23.8 after surgery, based on a study involving 9 patients [6]. 

In cases of joint disruption, the thickness and quality of the interposed tissue are crucial factors for achieving pain relief and functional improvement in terms of ROM [13, 14]. Therefore, the authors employed a combination of dorsal capsule tissue and dermal allograft to interposition sufficient tissue between the radiocarpal joint. In contrast to the traditional technique of folding the dermal allograft for insertion, they referred to Hartzler et al.‘s method [14] and utilized suture anchors for robust fixation to ensure secure anchoring of the ADM. Through this surgical approach, the authors achieved satisfactory results in terms of VAS score, DASH scores, ROM, and maintenance of the radiocarpal joint distance.

Four-corner arthrodesis may be considered for application in SNAC SLAC stages; however, it exhibits a higher complication rate and revision rate compared to PRC. Moreover, it poses limitations in cases of pre-existing radiolunate articular degeneration, making its application challenging in such scenarios [3, 15–17]. 

Recently, favorable outcomes have been reported with the use of resurfacing capitate pyrocarbon implants in cases of progressed radiocarpal arthritis. However, complications such as osteolytic changes, implant loosening, dislocation, or periprosthetic fractures have also been documented [18, 19]. 

In 2023, The results of a comparative analysis between 10 studies (147 cases) that underwent interposition arthroplasty after PRC and 8 studies (136 cases) that underwent capitate resurfacing. It showed a higher incidence of conversion to total wrist arthrodesis in the interposition group. On the other side, postoperative complications were reported to be more frequent in the capitate resurfacing group [20]. 

To successfully maintain long-term interposition arthroplasty, it is crucial to securely fix a sufficient amount of tissue to prevent bone-to-bone contact in the radiocarpal joint. The interposition, in this context, is essential for preventing complications associated with capitate resurfacing. We propose a method of interposition arthroplasty that combines the dorsal capsule and dermal allograft, aiming to enhance the effectiveness of conventional interposition arthroplasty without the complications of capitate resurfacing.

This study holds significance as it reports satisfactory clinical and radiological outcomes after performing interposition arthroplasty with a combination of dorsal capsule and dermal allograft following PRC in 14 patients.

### Limitation

Firstly, it is a non-randomized, retrospective level IV case study, which may introduce bias and limit the generalizability of the findings. Secondly, the sample size is relatively not much, with only 14 cases included, which may affect the statistical power and limit the ability to draw robust conclusions. Thirdly, the follow-up period is limited to one year, and a longer-term follow-up and randomized controlled study with a sufficient sample size would be beneficial to further evaluate the outcomes of the PRC with interposition arthroplasty using both capsular flap and ADM. Despite these limitations, this study holds significance as it reports satisfactory clinical and radiological outcomes after performing interposition arthroplasty with a combination of dorsal capsule and dermal allograft following PRC in 14 patients.

## Conclusion

The PRC with interposition arthroplasty using both capsular flap and ADM showed satisfactory clinical improvement, and the radiological results demonstrated the maintenance of a sufficient distance in the RCD after surgery.

## Data Availability

No datasets were generated or analysed during the current study.
